# The Initiation of GTP Hydrolysis by the G-Domain of FeoB: Insights from a Transition-State Complex Structure

**DOI:** 10.1371/journal.pone.0023355

**Published:** 2011-08-09

**Authors:** Miriam-Rose Ash, Megan J. Maher, J. Mitchell Guss, Mika Jormakka

**Affiliations:** 1 School of Molecular Bioscience, University of Sydney, New South Wales, Australia; 2 Structural Biology Program, Centenary Institute, Sydney, New South Wales, Australia; University of Queensland, Australia

## Abstract

The polytopic membrane protein FeoB is a ferrous iron transporter in prokaryotes. The protein contains a potassium-activated GTPase domain that is essential in regulating the import of iron and conferring virulence to many disease-causing bacteria. However, the mechanism by which the G-domain of FeoB hydrolyzes GTP is not well understood. In particular, it is not yet known how the pivotal step in GTP hydrolysis is achieved: alignment of a catalytic water molecule. In the current study, the crystal structure of the soluble domains from *Streptococcus thermophilus* FeoB (NFeoB*^St^*) in complex with the activating potassium ion and a transition-state analogue, GDP⋅AlF_4_^−^, reveals a novel mode of water alignment involving contacts with the protein backbone only. In parallel to the structural studies, a series of seven mutant proteins were constructed that targeted conserved residues at the active site of NFeoB*^St^*, and the nucleotide binding and hydrolysis properties of these were measured and compared to the wild-type protein. The results show that mutations in Thr35 abolish GTPase activity of the protein, while other conserved residues (Tyr58, Ser64, Glu66 and Glu67) are not required for water alignment by NFeoB*^St^*. Together with the crystal structure, the findings suggest a new mechanism for hydrolysis initiation in small G-proteins, in which the attacking water molecule is aligned by contacts with the protein backbone only.

## Introduction

The polytopic membrane protein FeoB is responsible for the import of ferrous iron (Fe^2+^) in prokaryotes [1], [2], [3], and is a major virulence factor in pathogenic bacteria such as *Camphylobacter jejuni* [4], *Legionella pneumophila* [5] and *Helicobacter pylori* [3]. The protein is separated into three domains: An N-terminal cytoplasmic GTPase domain (G-domain), a helical domain (together with the G-domain termed ‘NFeoB’), and a C-terminal polytopic membrane-spanning domain. While the import of Fe^2+^ by FeoB is dependent upon GTP hydrolysis by the G-domain [6], [7], the mechanism of coupling between the two events is unknown. The integrity of the G-domain is, however, instrumental in permitting bacterial colonization in anaerobic or acidic environments, such as the human gastrointestinal tract, where Fe^2+^ is the predominant form of iron.

Like other small G-proteins, the G-domain of NFeoB undergoes significant structural rearrangement in response to nucleotide binding and hydrolysis [8]. Two regions of the protein, termed Switch I and Switch II, adopt their active conformations upon GTP association and return to their inactive conformations after hydrolysis of GTP to GDP. Recently, it was shown that the rate of nucleotide hydrolysis and conformational switching by NFeoB is accelerated in the presence of potassium ions, and the protein has been classified as a member of the potassium-activated TrmE-Era-EngA-YihA-Septin-like (TEES) superfamily of G-proteins [8]. This superfamily is characterized by the presence of two invariant asparagine residues, which are predicted to be instrumental in creating a conserved potassium binding site in members of this family [8], [9].

Aside from its potassium-activated GTPase activity, very little is known about the mechanism of GTP hydrolysis by FeoB. In the hydrolysis reaction catalysed by all small G-proteins, nucleotide cleavage is initiated when a bound water molecule conducts a nucleophilic attack on the GTP terminal phosphate. However, while there is strict conservation of motifs responsible for nucleotide *binding* in G-proteins (G-motifs G1-G4), there is no single universal means by which these enzymes correctly position the attacking water molecule for catalysis. The mechanism by which G-proteins from the eukaryotic Ras-like family achieve water alignment has been thoroughly investigated, due in part to the oncogenicity of GTPase-deficient Ras G-protein mutants ([10] and references therein). For example, in Ras, Ran and Rho, the water is positioned by an invariant glutamine residue [10], [11], [12], while in Sar1 this function is instead performed by a histidine residue [13]. In these and other examples, the residues directly involved in catalysis have been identified both through site-directed mutagenesis, and through the determination of structures of the GTPases bound to both their activating proteins and transition-state analogues, such as GDP·AlF_x_, that capture the G-protein with all catalytic residues in their active conformations.

Sequence alignment shows that FeoB does not possess an obvious water-aligning residue in the equivalent position to that described above for Ras-like G-proteins [14]. Instead, the G-domain of FeoB has a hydrophobic amino acid in this position, which, in the various structures of NFeoB bound to a GTP analogue, is oriented away from the nucleotide binding site [7], [8], [15], [16], [17]. Such G-proteins have been termed “hydrophobic amino acid substituted GTPases” (HAS-GTPases) [14], and most prokaryotic G-proteins may be classed into this group [14], [18]. Given the importance of such prokaryotic GTPases in crucial cellular processes such as ribosome biogenesis [19], [20], DNA replication [19], and now virulence and iron transport, their hydrolysis mechanism has received surprisingly little attention and is still poorly understood. Only two have had their catalytic, water-aligning residue identified: MnmE [9] and YqeH [21]. In both cases, water alignment is achieved via an acidic residue from Switch II, however a lack of sequence conservation with other HAS-GTPases suggests that this is not a common mechanism of water alignment by G-proteins in this group.

Studying the mechanism of GTP hydrolysis by NFeoB will provide insight not only into the Feo iron transport system, but also on the wider family of HAS-GTPases. Earlier reports on the GTPase activity of FeoB have included some mutational analysis attempting to identify catalytically important residues [6], [16], however these studies were performed in the absence of the activating potassium and were unable to shed any light on the catalytic mechanism of FeoB. In the current work, we have explored the GTPase reaction of *Streptococcus thermophilus* NFeoB (NFeoB*^St^*) through an extensive mutational analysis and by crystallizing the protein in complex with the activating potassium and a transition-state analogue, GDP·AlF_4_^−^. The structure of the transition-state complex reveals an intriguing mode of water alignment by NFeoB*^St^*, in which the water is aligned solely through interactions with the protein backbone. Finally, with NFeoB structures having now been solved in *apo*-, GDP- and GTP-bound forms, the transition-state complex provides the final structural snapshot to complete our understanding of the conformational transitions associated with nucleotide hydrolysis in this important system.

## Results

### Overall structure of NFeoB*^St^* bound to GDP⋅AlF_4_^−^

NFeoB*^St^* was co-crystallized with the transition-state analogue, GDP⋅AlF_4_^−^. Crystals were obtained in space group *P*2_1_2_1_2_1_ with two molecules in the asymmetric unit, and the crystals diffracted to a resolution of 2.5 Å. Coordinates and structure factors have been deposited under PDB accession code 3SS8, and the data processing and refinement statistics are presented in Table 1. The final model contains residues 1–257 in both protein chains, and 72 water molecules. Each protomer is bound to GDP·AlF_4_^−^, one K^+^ ion, and one Mg^2+^ ion. The overall structures of both protein chains in the asymmetric unit agree very closely, and share an r.m.s. deviation of only 0.33 Å over all Cα atoms. Therefore, descriptions of the structural features of GDP·AlF_4_^−^-bound NFeoB*^St^* apply to both chains in the asymmetric unit, unless otherwise specified.

**Table 1:** Crystallographic data processing and refinement statistics.

Data processing	
Wavelength (Å)	0.95369
Space group	*P*2_1_2_1_2_1_
Unit cell dimensions	
*a, b, c* (Å)	48.3, 75.4, 156.5
Resolution (Å)	50–2.50 (2.54–2.50)
Total reflections	81,867
Unique reflections	20,085 (987)
Completeness (%)	99.5 (97.7)
〈*I*/σ(*I*)〉	10.4 (2.4)
〈Redundancy〉	4.1 (3.5)
*R*_merge_ a	0.122 (0.483)

Refinement	
No. molecules/asu	2
No. atoms	4100
No. water molecules	73
Resolution	50–2.51 (2.58–2.51)
Unique reflections	19,915 (1,333)
Completeness (%)	98.4 (91.0)
R.m.s.d bonds (Å)	0.010
R.m.s.d angles (°)	1.468
〈Protein B factor〉 (Å^2^)	27.2
Ramachandran plot^b^	
Favored (%)	97.5
Allowed (%)	100
*R*_work_ c	0.214
*R*_free_ d	0.252
PDB code	3SS8

Values in parentheses are for the highest resolution shell.

a *R*_merge_ = ∑_hkl_∑_j_ |*I*_j_(hkl)−〈*I*(hkl)〉|/∑_hkl_∑_j_ *I*_j_(hkl).

b As calculated by MolProbity [50].

c *R*_work_ = ∑_hkl_ |*F*_O_(hkl) – *F*_C_(hkl)|/∑_hkl_ |*F*_O_(hkl)|.

d Calculated as for *R*_work_, using 5% and of the diffraction data that was excluded during refinement.

**Figure 1:**
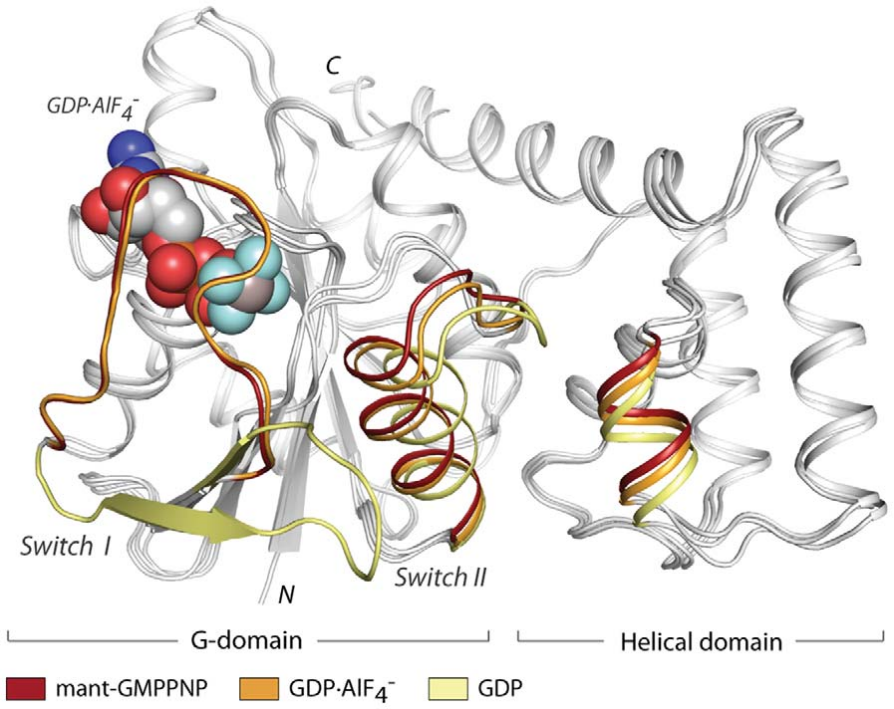
Overall structure of NFeoB*^St^* and the transitions associated with nucleotide hydrolysis. Superposition between NFeoB*^St^* structures bound to: a non-hydrolyzable GTP analogue mGMPPNP (red; 3LX5, [8]), GDP⋅AlF_4_^-^ (orange; this work), and GDP (yellow; 3LX8, [8]). The regions of the protein that undergo the largest structural change in response to nucleotide hydrolysis are shown in the colors listed above. The transition state analogue is illustrated in spheres.

The transition-state structure shows the canonical G-protein domain fold (residues 1–170) interacting with the helical domain (residues 171–270) (Figure 1). The helical domain is comprised of five helices, three of which interact with one face of the G-domain via a mixture of polar and hydrophobic contacts. For a thorough discussion of these interactions, readers are referred to a recent structural study upon NFeoB from *Thermotoga maritima*, in which a detailed analysis of the interface between the G- and helical domains is presented [7].

Superposition of the mGMPPNP-bound (a non-hydrolyzable GTP analogue), GDP⋅AlF_4_^−^-bound, and GDP-bound forms of NFeoB*^St^* show that the transition-state analogue has captured key regions of the protein in intermediate orientations between GTP- and GDP-bound states. As illustrated in Figure 1, conversion of the GTP substrate to its transition-state causes Switch II, and the adjacent portion of the helical domain, to shift away from the nucleotide binding site. Switch I, however, remains locked its active conformation, capping the nucleotide in the same orientation observed in the mGMPPNP-bound structure.

### Interactions with the activating potassium ion

**Figure 2:**
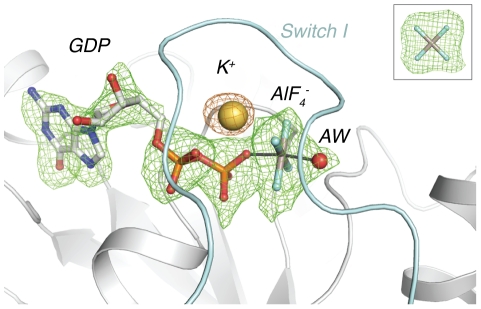
Omit and anomalous-difference Fourier maps at the nucleotide binding site. Shown in mesh are an *F*_O_ *-F*_C_ omit map about the GDP·AlF_4_^-^ transition state analogue and the attacking water molecule, and likelihood-based anomalous difference Fourier map illustrating the position of the potassium ion. The omit map is colored green, and the anomalous difference Fourier map is colored orange. Both maps are contoured to 3 σ, and Switch I is colored light blue. **Inset:** *F*_O_ *-F*_C_ omit map around the aluminofluoride, contoured at 3 σ.

In our previous work detailing the activation of NFeoB*^St^* by potassium, crystallization of the protein with the non-hydrolyzable GTP analogue mGMPPNP prevented co-crystallization with potassium, since the imido group from the nucleotide derivative obscured the predicted cation binding site. This problem has been circumvented in the transition-state structure, and clear *F*_O_ *-F*_C_ electron density could be observed for the potassium ion. In addition, at the data collection energy of 13 keV, there was sufficient anomalous scattering from the ion to observe an 8 σ peak in a likelihood-based anomalous difference Fourier map, which allowed unambiguous positioning of the potassium ion (Figure 2).

**Figure 3:**
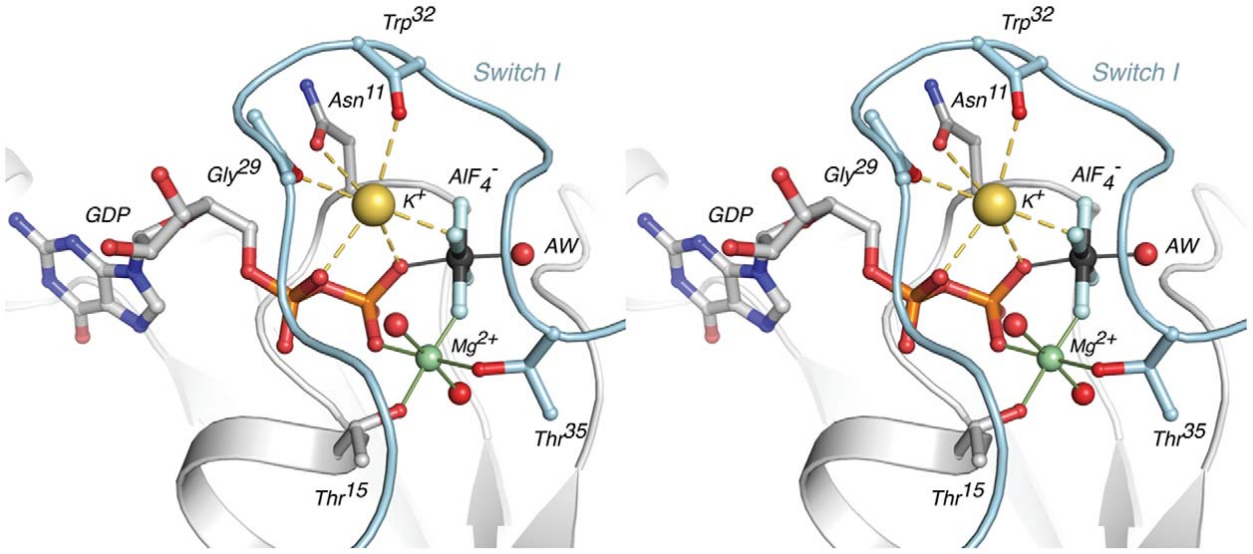
Stereo view of metal coordination spheres at the nucleotide binding site. The potassium ion is shown as a yellow sphere, the magnesium as a green sphere, and aluminum atom as a grey sphere. The attacking water is labeled *‘AW’.* Switch I is colored light blue. The interactions with the potassium ion, primarily electrostatic in nature, are shown as long dashes.

The potassium ion lies at the nucleotide binding site between the G1 P-loop and the apex of Switch I (Figure 3). The ligands for the potassium ion are the backbone carbonyls of Gly29 and Trp31, the sidechain amide oxygen of Asn11, one oxygen each from the β- and γ-phosphates, and one fluorine atom from the transition state analogue. This binding site confirms the results from our previous experiments, in which mutation of Asn11 abolished potassium binding and potassium-dependent GTPase activation [8]. The position of the ion overlays with that found in the potassium-bound structure of MnmE [9], providing yet more evidence for the suggestion that members of the TEES superfamily share a conserved potassium binding site [8], [9].

The transition-state structure now reveals why the GTPase activity of the protein is activated by potassium ions and not sodium ions. The bond lengths about the potassium are long, up to 3.2 Å, which is necessitated by the strict geometric constraints imposed by the protein and the nucleotide ligands (Figure S1). In this way, a binding site is created which is selective for potassium rather than sodium – a smaller ion that prefers significantly shorter metal-oxygen bond lengths. An analysis of high-resolution small molecule structures showed that the mean metal-oxygen distance at Na^+^ binding sites was 2.42 Å, with very few structures having bond lengths as long as those required at the cation binding site in NFeoB*^St^* (between 2.7 to 3.2 Å; Figure S1) [22]. Conversely, the preferred metal-oxygen bond distance for K^+^ was 2.84 Å, and unlike sodium, potassium binding sites were found to frequently accommodate bonds up to 3.4 Å in length [22]. Furthermore, the coordination sphere at the cation binding site in NFeoB*^St^* is highly distorted away from any regular geometry, precluding the binding of, for example, transition-metal cations that prefer strict directional covalency with short bond-lengths [22].

### Interactions with the attacking water and the transition-state analogue

The GDP nucleotide is bound to the protein via the well-described interactions typical of G-proteins (reviewed in [23]). Transition-state analogue formation using a mixture of GDP, AlCl_3_, and NaF is known to produce two species which both mimic the transition-state: GDP⋅AlF_3_ and GDP⋅AlF_4_^−^ [24], [25]. In the current structure, the *F*_O_-*F*_C_ electron density about the AlF_x_ moiety allowed unambiguous identification of the aluminofluoride as AlF_4_^−^ (Figure 2). The planar AlF_4_^−^ moiety sits 1.9 Å from the β-phosphate of the nucleotide, mimicking what is predicted to be the transition state structure of the γ-phosphate during hydrolysis. The AlF_4_^−^ group forms a number of contacts with the protein, the GDP nucleotide, and the magnesium atom (Figure 3). The aluminum atom is hexa-coordinated by the four fluorine atoms, one of the β-phosphate oxygens, and the nucleophilic attacking water molecule. The backbone amides from Switch I residues Gly33, Val34, and Thr35 hydrogen bond with two of the AlF_4_^−^ fluorine atoms, and Lys14 from the G1 motif provides an ionic interaction to neutralize some of the negative charge on the transition-state mimic.

**Figure 4:**
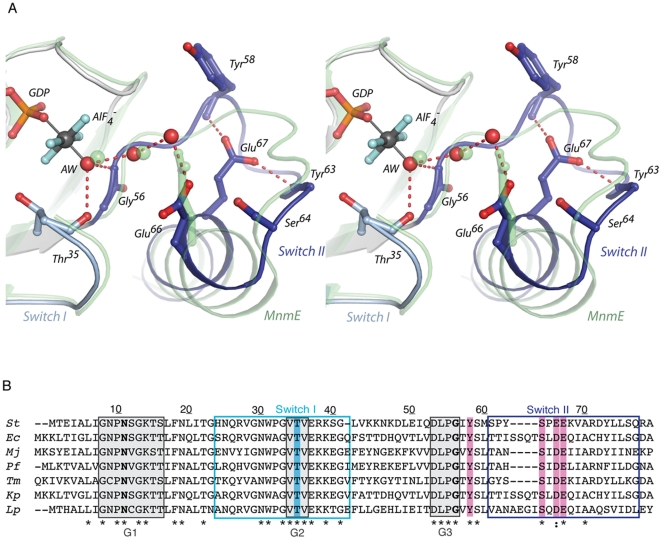
Primary sequence alignment and positions of conserved active site residues in the transition-state structure. (A) Stereo view illustrating the orientation of active site residues in chain A when bound to GDP·AlF_4_^−^. Switches I and II are colored in light and dark blue, respectively. Switch I residues 24–34 have been omitted for clarity. The structure of MnmE bound to GDP·AlF_4_^−^ (2GJ8, [9]) is overlayed, and is shown in transparent green cartoon. Its catalytic residue, Glu282, is shown as transparent sticks, and its active site waters as transparent green spheres. (B) Sequence alignment of FeoB from various organisms. Thr35 is highlighted in blue. Conserved residues in the vicinity of the GTP terminal phosphate that were mutated in the current study are highlighted in pink. Asn11 and Gly56 are shown in bold. Numbering is for the sequence from *S. thermophilus.* Abbreviations are as follows: *St*, *S. thermophilus* (Q5M586); *Ec, E. coli* (P33650); *Mj, Methanococcus jannaschii* (Q57986); *Pf, Pyrococcus furiosus* (Q8U2H8); *Tm, Thermotoga maritima* (Q9WXQ8); *Kp, Klebsiella pneumoniae* (C4X1R9); *Lp, Legionella pneumophilia* (Q8GNS3). Sequence alignment was performed using CLUSTALW2 [49].

The nucleotide binding site also features a magnesium ion, which is coordinated by one of the AlF_4_^−^ fluorine atoms, an oxygen from the β-phosphate, two *trans* waters, and residues Thr15 and Thr35 from the G1 and G2 motifs, respectively (Figure 3). Thr35 is a fully conserved residue in FeoB, and its coordination to the magnesium ion in the transition-state structure is in agreement with structural and functional studies on other small G-proteins (reviewed in [26]). In its magnesium-bound conformation, the backbone carbonyl of Thr35 hydrogen-bonds with the attacking water molecule, which is also the recipient of another hydrogen bond from the backbone amide from Gly56 from the G3 motif (Figure 4A). The importance of these hydrogen bonds will become apparent in the context of the mutational analysis described later.

### The role of Thr35 and Switch I in nucleotide hydrolysis

Despite the clear involvement of Thr35 in magnesium binding in the current structure, a host of earlier NFeoB structures exhibited disordered Switch I loops in their GTP-bound forms (2WIC [15], 3HYT [17] and 2WJI [16]), or possessed GTP-bound Switch I loop orientations that were unchanged from their GDP-bound conformation (3A1U [7], 3I92 (unpublished)). In all these structures, the G2 threonine was not coordinated to the magnesium atom. It was therefore suggested that FeoB was an atypical G-protein, in which the G2 threonine was not involved in magnesium binding [7], [15], [16]. However, in light of the current structure providing evidence otherwise, we have probed the role of this residue in GTP hydrolysis by NFeoB*^St^* through the construction of two point mutants: T35A and T35S. For both mutant constructs, and for other mutants described later, both the basal and activated rates of GTP hydrolysis were measured. We consider basal, intrinsic hydrolysis rates to be those measured in the presence of NaCl, while activated rates are those measured in KCl. By separately determining the two rates, we may distinguish between mutations which affect the overall hydrolysis reaction, and those that affect activity by interfering with the potassium binding site only. Finally, in order to determine whether nucleotide binding was impaired in any of the mutant proteins, the rate of mant-GTP binding and mant-GDP release were also examined.

**Figure 5:**
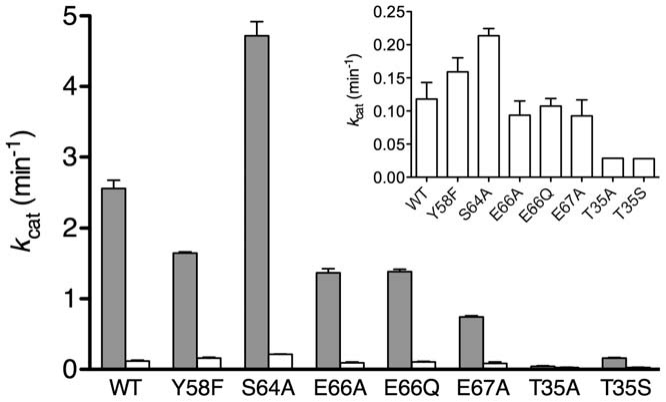
GTPase activities of NFeoB*^St^* mutants. Grey bars indicate the GTPase activity in KCl, and white bars (including inset) are rates in NaCl. Values are the mean from at least three independent measurements with errors bars indicating standard deviations. The wild-type rates are from a previous study [8].

For both T35A and T35S, we found that the basal level of GTPase activity was essentially abolished in these mutants, being <0.04 min^−1^ and more than three-fold lower than the wild-type value of 0.12 min^−1^ (Figure 5 and Table 2). In addition, the rate of association with mant-GTP was decreased more than three-fold in the NFeoB*^St^* T35A mutant, signaling disruption of the native GTP binding site. The rate of mant-GDP release, however, remained unaffected, in keeping with the numerous structures of GDP-bound NFeoB in which Thr35 is distal from the active site and does not interact with the nucleotide [7], [8], [15], [16]. The decrease in intrinsic GTPase activity of the Thr35 mutants are in agreement with studies on Ras and Gsα, in which mutation of the G2 threonine to alanine resulted in 4- and 20-fold reductions in GTPase activity, respectively [27], [28].

**Table 2:** Nucleotide binding and hydrolysis properties of NFeoB*^St^* mutants.

	k_cat_ (min^−1^) NaCl	k_cat_ (min^−1^) KCl	k_obs_ (s^−1^) mant-GTP	k_off_ (s^−1^) mant-GDP
Wild-type	0.12±0.02	2.6±0.2	24.0	14.1
T35A	<0.04	<0.04	6.8	13.7
T35S	<0.04	0.16±0.02	31.1	14.4
Y58F	0.16±0.02	1.65±0.03	25.2	n.d.^a^
S64A	0.21±0.01	4.7±0.3	27.3	15.8
E66A	0.09±0.02	1.4±0.1	19.6	12.1
E66Q	0.11±0.01	1.39±0.06	18.6	10.8
E67A	0.09±0.02	0.72±0.06	25.7	10.2

Activity measurements were performed under multiple-turnover conditions in the presence of either NaCl or KCl. For both T35S and T35A in NaCl and for T35A in KCl, *k*_cat_ values were too low to be accurately determined above background. Wild-type values are from an earlier study [8]. Values are given as the mean from at least three independent measurements, with errors quoted as standard deviations.

a Not determined.

Even in the presence of the activating potassium, the GTPase activity of the T35A mutant was similarly abolished below the intrinsic level. This suggests that due to the loss of magnesium coordination by Thr35, the potassium binding site, formed by the apex of Switch I (Figure 3), has been disrupted in this mutant. The more conservative T35S mutation, however, showed modest but unmistakable potassium-dependent activation (Figure S2), although its maximal hydrolysis rate was still 16-fold lower than the equivalent rate in wild-type protein (Figure 5 and Table 2). The retention of some potassium-dependent activation in T35S shows that the presence of a serine residue at this position can at least partially restore magnesium coordination by Switch I, the adoption of the native Switch I structure, and the formation of the potassium binding site.

The biochemical characterization of the T35S and T35A mutant proteins highlights the importance of magnesium coordination by Thr35 not only for GTP binding, but also for the efficacy of the subsequent hydrolysis reaction. Furthermore, the transition-state structure reveals why the loss of magnesium coordination is so detrimental to GTPase activity. As illustrated in Figure 4A, the backbone carbonyl of Thr35 hydrogen-bonds with the attacking water molecule. Magnesium binding by Thr35 therefore not only positions Switch I to create the potassium binding site, but it also correctly orients the protein backbone to assist in aligning the catalytic water molecule for its nucleophilic attack. This hydrogen bond was similarly suggested to have important implications for water alignment and the hydrolysis mechanism of Gsα proteins [28].

### A comparative study with MnmE

While the crucial interaction between the backbone carbonyl from the G2 threonine and the attacking water molecule is common to all small G-proteins, previous studies have shown that additional water-aligning residues, such as the catalytic glutamine from Ras, are needed to attain maximal GTPase activity (reviewed in [29]). Recently, the structural similarity between the G-domains of MnmE and NFeoB*^St^* prompted the recognition that FeoB is a member of the potassium-activated TEES family of G-proteins, to which MnmE also belongs [8], [9]. Both proteins are also HAS-GTPases, and possess a hydrophobic residue in place of the catalytic glutamine from the Ras-GTPases. In MnmE, the water-aligning residue was identified as Glu282, which is part of the Switch II helix (Figure 4A) [9]. Similarly, the catalytic residue from YqeH, another TEES- and HAS-GTPase, was found by mutational studies to be Asp57, and this residue was predicted by homology modeling to lie in the equivalent position as Glu282 from MnmE [21].

Superposition of the transition-state complex structures of MnmE and NFeoB*^St^* shows that Glu282 overlays with Glu66 from NFeoB*^St^* (Figure 4A). This position is always conserved as an acidic amino acid in FeoB (Figure 4B), and the equivalent residue in the full-length *E. coli* protein has been shown to be essential for *in vivo* iron transport [6]. Together, these observations raise the possibility that Glu66 might be a crucial residue for initiating catalysis in NFeoB proteins, in a similar manner as Glu282 from MnmE and Asp57 from YqeH. In the crystal structure of GDP⋅AlF_4_^−^-bound NFeoB*^St^*, Glu66 exhibits two conformations: in chain A, there is weak electron density signaling at least its partial occupancy of the conformation shown in Figure 4A, which, relative to the GTP-bound structure, is a re-orientation toward the nucleotide binding site. Conversely, in chain B, the Glu66 residue remains oriented away from the nucleotide.

However, there is one principal difference between the active sites of MnmE and NFeoB*^St^*. In MnmE, Glu282 aligns the attacking water via a bridging water molecule. Yet while the attacking water and the bridging water overlay well between the two structures, the carboxylate oxygen from Glu282 in MnmE has been replaced by a third water molecule in NFeoB*^St^* (Figure 4A). While this third water *does* hydrogen-bond with the sidechain of Glu66 in its chain A orientation, the increased length of the water chain raises the question as to whether Glu66 is indeed involved in water alignment by NFeoB*^St^*, or whether the protein has adapted a different means of positioning the attacking water molecule for catalysis.

### Functional characterization of Switch II mutants

In order to investigate the possible role of Glu66 in the hydrolysis mechanism of NFeoB*^St^*, two mutant proteins were constructed: E66A and E66Q. In addition, an alignment of FeoB sequences shows that there are several other conserved residues in Switch II (Figure 4B), three of which are in the vicinity of the terminal phosphate in the structure of mGMPPNP-bound NFeoB*^St^* (Figure S3). These residues, Tyr58, Ser64, and Glu67, could also potentially be involved in the catalytic cycle of FeoB. Therefore, three further mutant proteins were constructed: Y58F, S64A and E67A. For all mutants, GTPase activities and nucleotide binding properties were characterized as for the Thr35 mutant constructs.

Mutation of Tyr58, Ser64, Glu66, and Glu67 did not affect the rate at which mant-GTP associated with the protein nor the rate at which mant-GDP dissociated from the protein (Table 2), showing that nucleotide binding and release were not impaired in any of the mutant constructs. The greatest decrease in potassium-activated activity was only 3.5-fold, which arose from the E67A mutant (Figure 5 and Table 2). Therefore, no mutation affected GTPase activity to the magnitude that would be expected should these residues play a pivotal role in aligning the attacking water molecule and initiating catalysis. For example, the E282A mutation in MnmE caused a loss of potassium-activated GTPase activity by more than three orders of magnitude [9], and mutation of the catalytic gluatmine in Ras decreased p120-GAP activated GTPase activity by 10^5^-fold [30].

It thus appears that Tyr58, Ser64, Glu66, and Glu67, despite their conservation and proximity to the nucleotide γ-phosphate, do not play an essential role in water alignment in NFeoB*^St^*. This is further supported by the transition-state structure, in which the orientations of Tyr58, Ser64, and Glu67 remain unchanged relative to their GTP-bound conformations and do not interact with the attacking water (Figure S3 and Figure 4A).

Therefore, the transition-state structure and the functional characterization of the Switch II mutants together imply that the attacking water is aligned not by any amino acid sidechain from NFeoB*^St^*, but rather through a different means. As noted earlier, there the protein does, in fact, form two hydrogen bonds with the attacking water molecule, albeit not via any conserved sidechain. These hydrogen bonds arise from the backbone amides of Thr35 and Gly56 (Figure 4A), and together with the mutational analysis, suggest that unlike in MnmE and YqeH, water alignment in NFeoB*^St^* is achieved by contacts with the protein backbone alone.

## Discussion

In the current study, we have presented the structure of *S. thermophilus* NFeoB bound to the transition-state analogue GDP⋅AlF_4_^−^. The structure confirmed the location of the potassium ion binding site, and revealed how the long bond lengths imposed upon the ion lead to selectivity in the cation-dependent activation of NFeoB*^St^*. The location of the ion matches that observed in MnmE [9], and provides further evidence in support of the hypothesis that members of the TEES superfamily of G-proteins possess a conserved potassium binding site [8], [9]. The nature of the structural basis for cation selectivity by NFeoB*^St^* may therefore also be extended to other members of the TEES superfamily, whose GTPase activity is similarly activated in the presence of potassium ions but not sodium ions [9], [21], [31], [32], [33].

Previous studies on the G-domain of FeoB have proposed that Thr35, although being essential for all other GTPases, is not important for magnesium coordination, GTP binding, and GTP hydrolysis by NFeoB [7], [15], [16]. However, the current transition-state structure provides evidence otherwise, showing that Thr35 is indeed involved in both magnesium coordination and alignment of the attacking water molecule via a hydrogen bond from its backbone carbonyl group. This was supported by the mutational analysis, in which mutation of Thr35 abolished intrinsic GTPase activity, suggesting that the attacking water molecule was not correctly aligned in the mutant protein. Furthermore, the loss of potassium-dependent activation in T35A showed that magnesium coordination by Thr35 is essential in conferring the GTP-bound Switch I conformation in NFeoB*^St^*, and creating the potassium binding site from the apex of the Switch.

Years of comprehensive studies into small G-proteins have demonstrated that there is no single universal means by which these enzymes poise the nucleophilic water molecule for its in-line attack on the GTP γ-phosphate. It has therefore become increasingly apparent that catalytically important residues in different G-proteins must be identified on a case-by-case basis. In the current study, the following seemingly paradoxical result has emerged: despite having an appreciable GTPase activity (*k*_cat_ = 2.6 min^−1^, [8]), NFeoB*^St^* does not appear to possess a catalytic, water-aligning residue. In the transition-state structure, no amino acid sidechain directly interacted with the attacking water molecule, and mutation of all conserved residues in the vicinity of the terminal phosphate failed to significantly diminish *in vitro* GTPase activity.

Some insight, however, may be gained from examining these results in the context of related G-proteins. The potassium-activated GTPase activity of wild-type NFeoB*^St^*, 2.6 min^−1^, is comparable to the activated rates from all other members of the TEES family whose activities have been measured. These include EngA, Era, YqeH, and YchF, which have activities between 0.1–2 min^−1^ [21], [31], [32], [33], [34], [35], [36]. MnmE from *E. coli* has the highest rate of GTP hydrolysis in the family, with a potassium-activated *k*_cat_ of 47 min^−1^ [9]. Importantly, when the catalytic residues in YqeH and MnmE were mutated, GTPase activity was diminished by several orders of magnitude, giving activities of <0.0001 min^−1^ and 0.025 min^−1^, respectively [9], [21]. The same is true for GTPases from other families, where activity was abolished by mutation of, for example, catalytic glutamine residues [30], or in the absence of catalytic residues provided in *trans* [37], [38], [39]. In these examples, GTPase activities dropped below 0.00035 min^−1^.

Based upon this wealth of data from other G-proteins, we may therefore make the following key observation: the appreciable hydrolysis rates of wild-type NFeoB*^St^* and its mutant derivatives are consistent with a GTPase that possesses all necessary active site residues. That is, should the protein be unable to align the attacking water molecule efficiently, we would expect GTPase activity to be several orders of magnitude lower than that which has been measured for NFeoB*^St^*. Therefore, this supports the results from the current study, which suggests that water alignment in NFeoB*^St^* is achieved only via hydrogen-bonding interactions with the backbone amides from Thr35 and Gly56, the conserved glycine residue from the G3 motif. Water alignment by the G3 glycine has also been proposed for the bacterial HAS-GTPase SsGBP [40], and attempts to locate a catalytic residue in YchF, another TEES- and HAS-NTPase, have likewise proved unfruitful [33]. Therefore, the current study adds to an increasing body of evidence that suggests this family of G-proteins does not possess a conserved means of water alignment, and in some cases, might align the attacking water through backbone contacts alone.

With regards to the FeoB protein, we should not, of course, exclude the possibility that GTPase activity in full-length FeoB is even further enhanced by insertion of an as yet unidentified residue into the active site. Such a residue might create yet another interaction with the attacking water molecule, or provide a favourable electrostatic interaction that increases GTPase activity of the protein. This residue could arise in *trans* from FeoA, a soluble protein that is co-expressed with FeoB in 80% of bacterial strains that possess the Feo iron transport system [41], or perhaps from a cytoplasmic loop from the membrane domain. Indeed, examination of the transition-state structure of NFeoB*^St^* shows that there is a cavity at the active site into which a catalytic residue might be inserted (Figure S4). Given that the C-terminus of the helical domain is on the same face of NFeoB as the active site, such a scenario is at least stereochemically plausible when the orientation of NFeoB relative to the membrane domain is considered (Figure S4). However, these and other hypotheses can only be tested when full-length FeoB can be fully characterized. In the same vein, the current results also highlight the need to now study the role of, for example, Glu66 in the context of the full-length protein, since this residue is essential for iron transport [6] and yet as we have now demonstrated, it is not involved in the GTPase reaction or GTP binding in the soluble domains alone.

The results from the current study have provided insight into the wider family of G-proteins to which the G-domain of FeoB belongs, highlighted the importance of Thr35 and Switch I in nucleotide hydrolysis by FeoB, and raised some interesting questions for further study into this interesting G-protein-coupled system.

## Materials and Methods

### Protein preparation

Wild-type NFeoB from *S. thermophilus* (residues 1–270; strain LMG 18311) in a pGEX-4T-1 GST fusion vector was expressed and purified as described [8]. Briefly, GST-tagged NFeoB*^St^* in 100 mM NaCl, 20 mM Tris pH 8.0 was passed over glutathione sepharose resin (GE Healthcare Life Sciences). The GST moiety was removed by thrombin cleavage overnight, and untagged NFeoB*^St^* allowed to elute from the beads. Pure NFeoB*^St^* was obtained after gel filtration chromatography on a Superdex 75 size exclusion column (GE Healthcare Life Sciences). The eluted protein was concentrated up to ∼10 mg/mL as determined by the BCA assay method. Protein aliquots could be flash-frozen and stored at −80°C without loss of enzyme activity. All point mutations (T35A, T35S, Y58F, S64A, E66A, E66Q and E67A) were generated using the Quikchange II Site-Directed Mutatgenesis Kit (Stratagene), and were purified as described for the wild-type protein. The sole exception was the S64A mutant protein, which appeared to be particularly sensitive to non-native disulfide bond formation after cell lysis. This mutant was purified to homogeneity with the addition of 10% glycerol and 5 mM DTT in all buffers.

### Crystallization

Crystallization conditions were screened at 20°C in 96-well trays (Greiner Bio-One) using the hanging-drop vapour diffusion method with commercially available screens (PACT, JCSG+ and Classics Suites; Qiagen). All crystal trays were set-up using a Mosquito nanoliter liquid handling robot (Molecular Dimensions), and drops contained equal volumes (150 µL) of reservoir and protein solution. To form the NFeoB*^St^*-GDP·AlF_x_ transition-state analogue complex for crystallization, wild-type NFeoB*^St^* was first exchanged into buffer containing 400 mM KCl and 20 mM Tris pH 8.0 using a Vivaspin 500 concentrator (10 kDa MWCO; GE Healthcare Life Sciences). After concentrating the protein to 5 mg/mL, the transition-state complex was formed by the addition of 10 mM MgCl_2_, 0.5 mM GDP, 5 mM NaF, and 0.5 mM AlCl_3_ to the protein solution. NFeoB*^St^* bound to GDP·AlF_x_ crystallized in condition C4 from the PACT screen, which contained 0.1 M PCB buffer pH 7.0, 25% PEG1500. Crystals grew within three days to approximate dimensions 20×20×200 µm, and were flash-frozen in liquid nitrogen after brief immersion in cryoprotectant solution containing mother liquor plus 20% glycerol.

### Data collection and refinement

Diffraction data was collected on beamline MX2 at the Australian Synchrotron [42], and was recorded on an ADSC Quantum 315r detector. Data was processed and scaled using the HKL-2000 suite [43], and molecular replacement conducted using the program *Phaser* [44] from the CCP4 program suite [45]. The model of wild-type NFeoB*^St^* bound to mant-GMPPNP (mGMPPNP; PDB code 3LX5) was used as a molecular replacement search model after removal of all non-protein atoms. Refinement was carried out using Refmac 5.5.0109 [46], and manual model building was performed in *Coot* [47]. Anomalous difference Fourier maps were constructed using *Phaser,* using the final model as partial structural information for SAD experimental phasing. All structure Figures were generated using MacPyMOL (The PyMOL Molecular Graphics System, Schrödinger, LLC) and structural superpositions performed using LSQKAB [48].

### GTPase assays

All hydrolysis measurements in the current study were conducted under steady-state conditions using a colorimetric assay to detect free phosphate. GTPase assays were carried out at 37°C in buffer containing 20 mM Tris pH 8.0, 5 mM MgCl_2_, and either 200 mM NaCl or 200 mM KCl. GTP was present at 350 µM for all assays, except those involving mutants T35S and T35A. Here, the GTP concentration was increased to 1 mM. Phosphate liberated from the GTPase reaction was measured using the Malachite Green Phosphate Detection kit (BioAssay Systems). Aliquots were removed at intervals appropriate to the rate of the reaction, and mixed in a 4∶1 ratio with the Malachite Green Reagent. For T35A and T35S in which GTP was present at a high concentration, aliquots were diluted 1∶4 before analysis. Colour was allowed to develop for 30 mins at room temperature, then the absorbance measured at 620 nm in a 96-well plate (Greiner Bio-One) on a POLARstar Omega microplate reader (BMG LABTECH). The *k*_cat_ was calculated from the slope of each time course. All assays were conducted a minimum of three times, using replicate protein concentrations between 0.3 and 1.0 µM.

### Stopped-flow experiments

Stopped-flow experiments to measure *k*_obs_ for mant-GTP and *k*_off_ of mant-GDP were preformed as described [8], [17] in buffer containing 20 mM Tris pH 8.0, 100 mM KCl, and 10 mM MgCl_2_. Briefly, *k*_obs_ was measured by the rapid mixing of an equal volume of 10 µM NFeoB*^St^* with 1 µM mant-GTP (Invitrogen). The rate of dissociation of mant-GDP (Invitrogen) from NFeoB*^St^* was measured through a competition experiment, in which 10 µM NFeoB*^St^* was incubated with 0.5 µM mant-GDP, then the solution rapidly mixed with an equal volume of 1 mM of unlabeled GTP (Sigma Aldrich). The mant fluorophore was excited at 355 nm, and fluorescence collected from all wavelengths shorter than 400 nm. All experiments were performed 7–10 times, and the data averaged before calculating *k*_obs_ or *k*_off_ values using first-order exponential functions.

## Supporting Information

Figure S1 **Selectivity at the cation binding site in NFeoB** ***^St^*** **.** All bond lengths are given in ångströms. Bonds to the potassium ion in the transition-state structure of NFeoB*^St^* are shown by yellow dashes. Red dashes show the predicted bond to the γ-phosphate when the protein is bound to GTP, before hydrolysis begins. Small black spheres indicate the ideal bond lengths for a sodium ion (2.42 Å), should it be positioned as for the potassium ion. Even when the overall coordinate error of the NFeoB*^St^* structure is considered (0.2 Å as estimated by Maximum Likelihood), the bond lengths at the cation binding site are much longer than those preferred for a sodium ion.
(TIF)

Figure S2 **GTP hydrolysis by T35A and T35S NFeoB** ***^St^*** **mutants.** T35A (circles) or T35S (triangles) were added to solutions containing GTP and either NaCl (open symbols) or KCl (closed symbols). The amount of phosphate liberated at each time point was determined using a colorimetric assay to detect free phosphate. Phosphate concentrations have been adjusted for the background at 0 min.
(TIF)

Figure S3 **Active site overlay between GTP-bound and GDP⋅AlF_4_^-^-bound structures of NFeoB** ***^St^*** **.** The structure of NFeoB*^St^* bound to a GTP-analogue, mant-GMPPNP, is colored green (3LX5), and the current transition-state structure (chain A) is colored blue. Active site waters are shown as opaque red spheres for the transition-state structure, and transparent red spheres for the mant-GMPPNP-bound structure. The hydrogen-bonding network involving the active site waters are shown as dashes. Switch I residues 24–34 from both structures have been removed for clarity, as has the disordered mant group from the nucleotide in the mant-GMPPNP-bound structure.
(TIF)

Figure S4 **Position of the catalytic cavity relative to the C-terminus of NFeoB** ***^St^*** **.** NFeoB*^St^* possesses a cavity at the active site into which a catalytic residue might be inserted. The G-domain is shown as surface, with the catalytic cavity outlined in yellow. The attacking water is shown as a red sphere. The helical domain is represented in cartoon, with its directionality indicated by transition from yellow to red. The position of the C-terminus of is indicated, demonstrating that in the context of the full-length protein, the catalytic cavity could potentially interact with a cytoplasmic loop from the membrane domain.
(TIF)
