# Supplementary figures and images for: The Initiation of GTP Hydrolysis by the G-Domain of FeoB: Insights from a Transition-State Complex Structure
Source: PLoS One. 2011 Aug 9;6(8):e23355. doi: 10.1371/journal.pone.0023355 (PMC3153494; doi:10.1371/journal.pone.0023355)

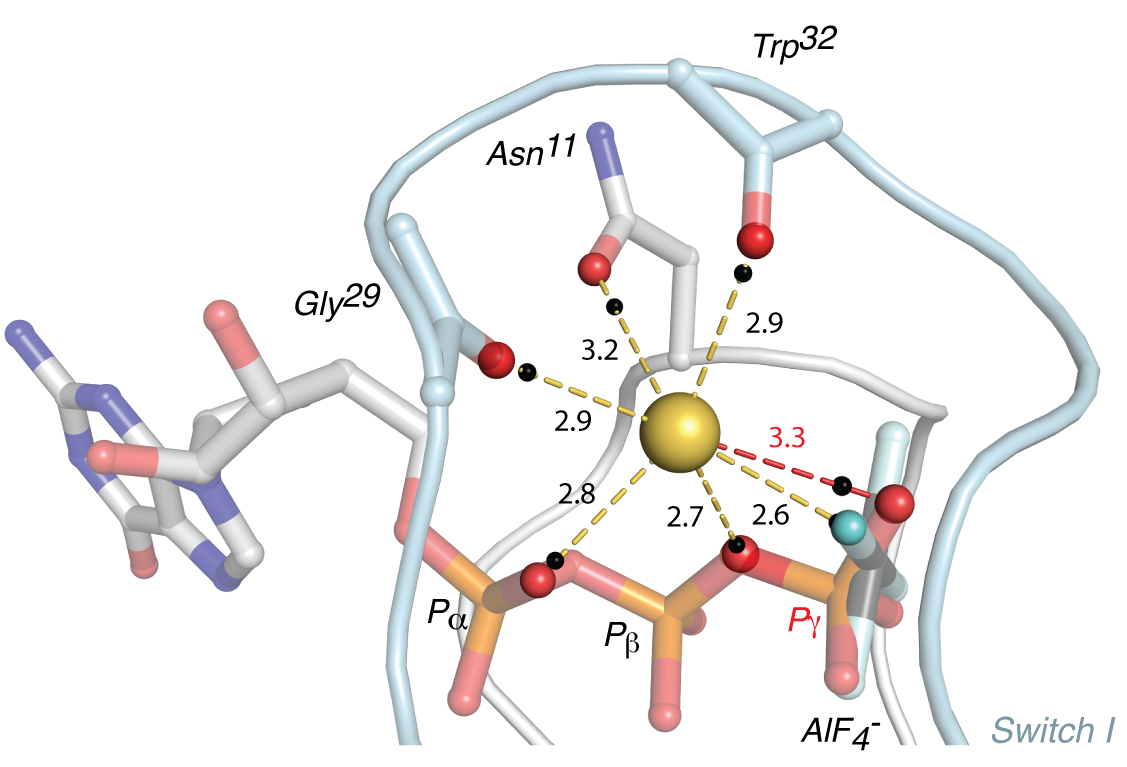

Supplement: Figure S1 — Selectivity at the cation binding site in NFeoB St . All bond lengths are given in ångströms. Bonds to the potassium ion in the transition-state structure of NFeoBSt are shown by yellow dashes. Red dashes show the predicted bond to the γ-phosphate when the protein is bound to GTP, before hydrolysis begins. Small black spheres indicate the ideal bond lengths for a sodium ion (2.42 Å), should it be positioned as for the potassium ion. Even when the overall coordinate error of the NFeoBSt structure is considered (0.2 Å as estimated by Maximum Likelihood), the bond lengths at the cation binding site are much longer than those preferred for a sodium ion. (TIF) [file pone.0023355.s001.tif]

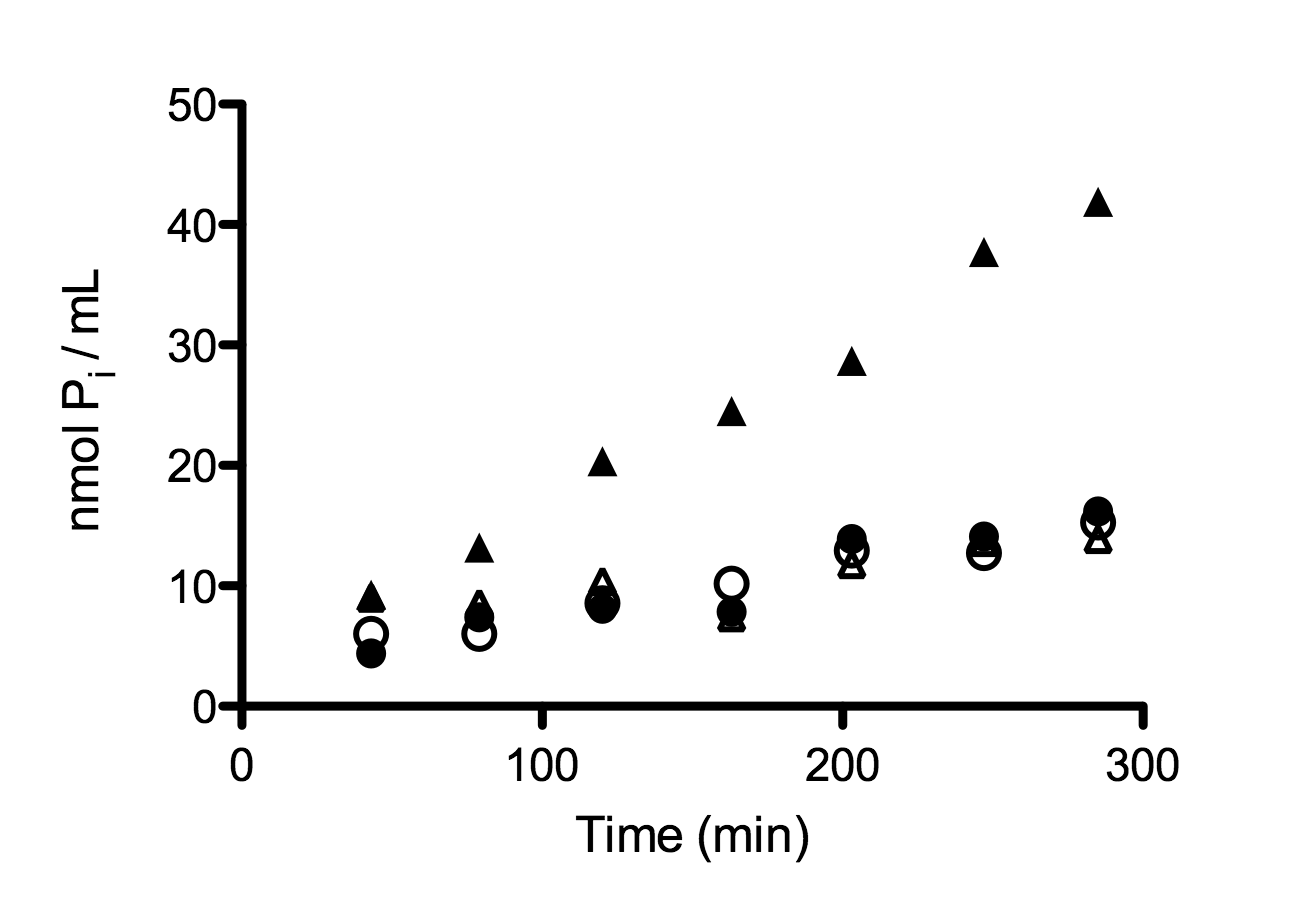

Supplement: Figure S2 — GTP hydrolysis by T35A and T35S NFeoB St mutants. T35A (circles) or T35S (triangles) were added to solutions containing GTP and either NaCl (open symbols) or KCl (closed symbols). The amount of phosphate liberated at each time point was determined using a colorimetric assay to detect free phosphate. Phosphate concentrations have been adjusted for the background at 0 min. (TIF) [file pone.0023355.s002.tif]

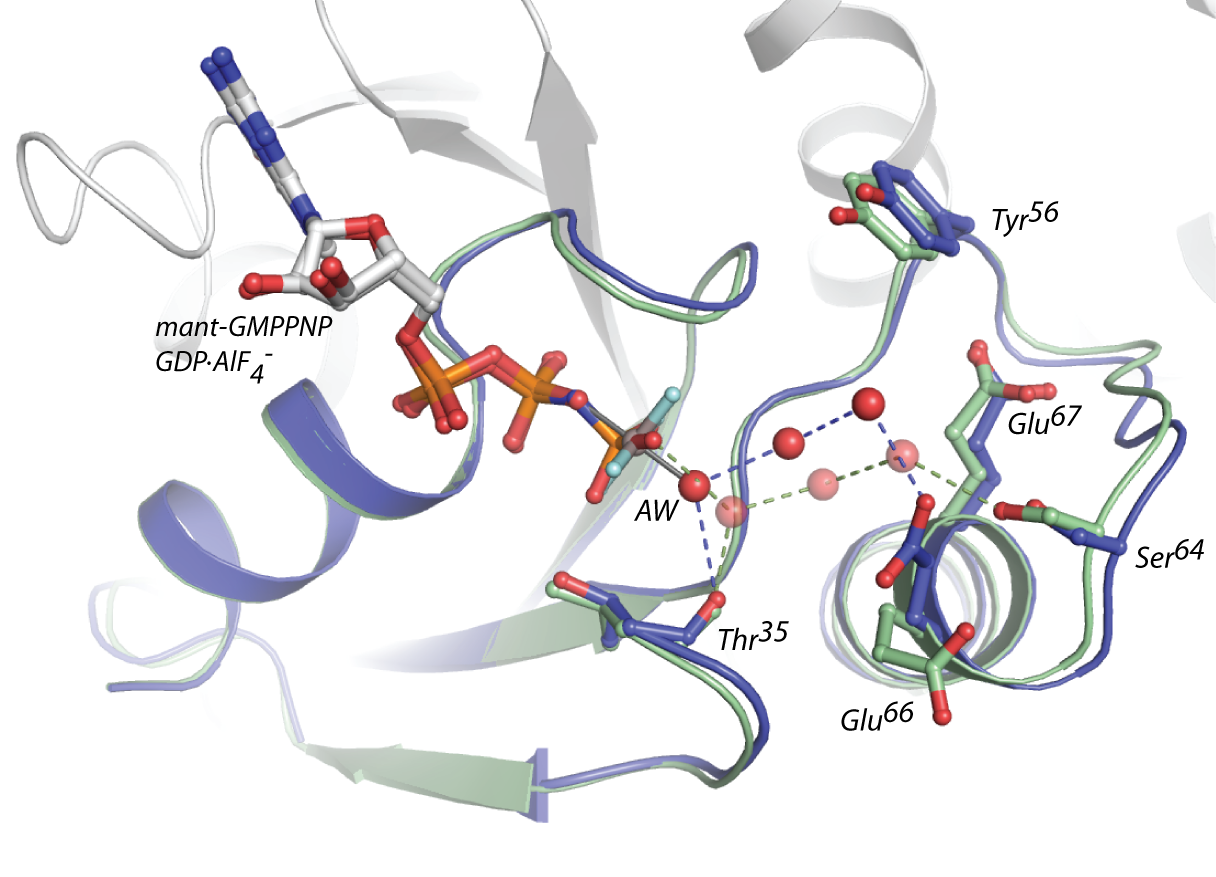

Supplement: Figure S3 — Active site overlay between GTP-bound and GDP⋅AlF4--bound structures of NFeoB St . The structure of NFeoBSt bound to a GTP-analogue, mant-GMPPNP, is colored green (3LX5), and the current transition-state structure (chain A) is colored blue. Active site waters are shown as opaque red spheres for the transition-state structure, and transparent red spheres for the mant-GMPPNP-bound structure. The hydrogen-bonding network involving the active site waters are shown as dashes. Switch I residues 24–34 from both structures have been removed for clarity, as has the disordered mant group from the nucleotide in the mant-GMPPNP-bound structure. (TIF) [file pone.0023355.s003.tif]

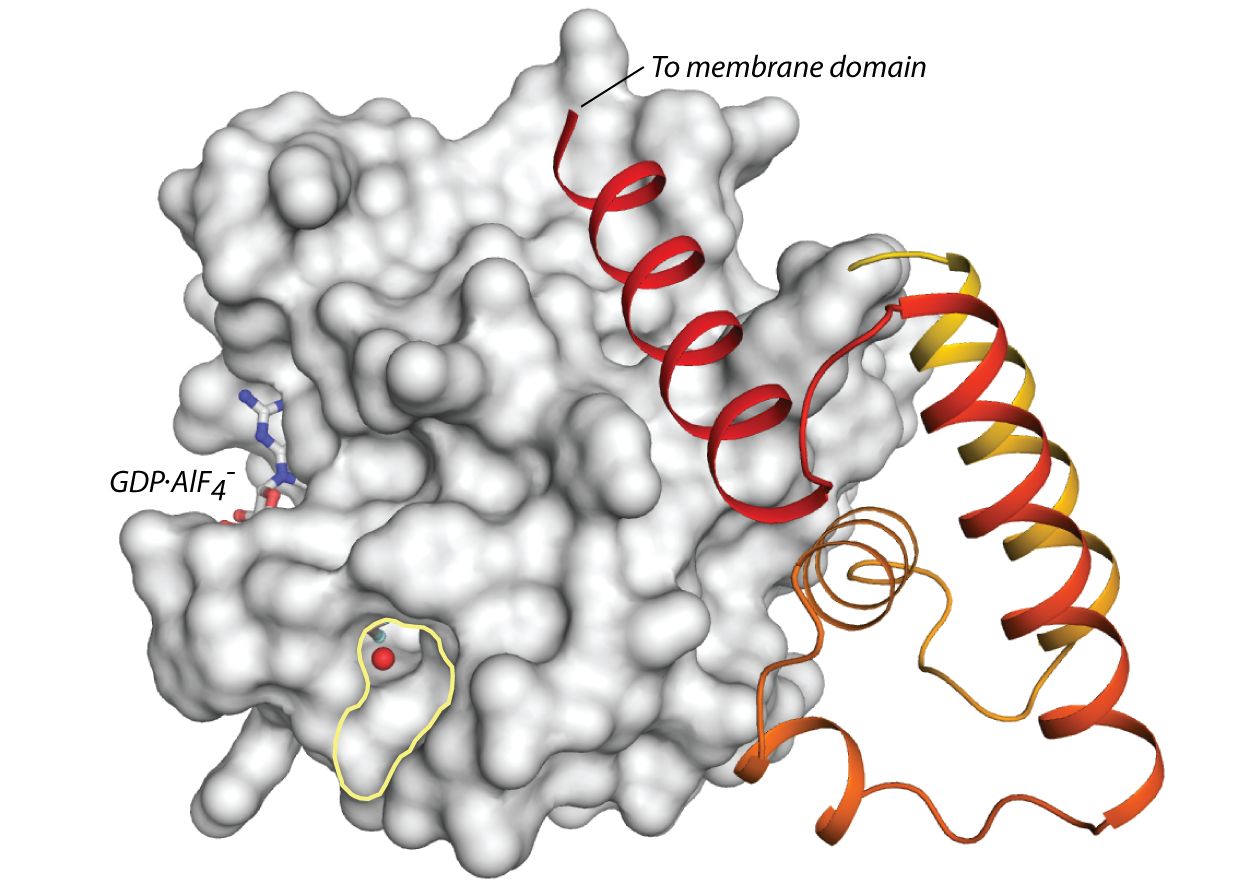

Supplement: Figure S4 — Position of the catalytic cavity relative to the C-terminus of NFeoB St . NFeoBSt possesses a cavity at the active site into which a catalytic residue might be inserted. The G-domain is shown as surface, with the catalytic cavity outlined in yellow. The attacking water is shown as a red sphere. The helical domain is represented in cartoon, with its directionality indicated by transition from yellow to red. The position of the C-terminus of is indicated, demonstrating that in the context of the full-length protein, the catalytic cavity could potentially interact with a cytoplasmic loop from the membrane domain. (TIF) [file pone.0023355.s004.tif]
